# Black-Si as a Photoelectrode

**DOI:** 10.3390/nano10050873

**Published:** 2020-05-01

**Authors:** Denver P. Linklater, Fatima Haydous, Cheng Xi, Daniele Pergolesi, Jingwen Hu, Elena P. Ivanova, Saulius Juodkazis, Thomas Lippert, Jurga Juodkazytė

**Affiliations:** 1 School of Science, RMIT University, Melbourne, VIC 3000, Australia; elena.ivanova@rmit.edu.au; 2 Melbourne Centre for Nanofabrication, ANFF, 151 Wellington Road, Clayton, VIC 3168, Australia; 3 Optical Sciences Centre and ARC Training Centre in Surface Engineering for Advanced Materials (SEAM), School of Science, Swinburne University of Technology, Hawthorn, VIC 3122, Australia; jhu@swin.edu.au (J.H.); sjuodkazis@swin.edu.au (S.J.); 4 Laboratory for Multiscale Materials Experiments, Paul Scherrer Institut, Villigen PSI, CH-5232 Villigen, Switzerland; haydous@kth.se (F.H.); xi.cheng@psi.ch (C.X.); Daniele.Pergolesi@psi.ch (D.P.); 5 Tokyo Tech World Research Hub Initiative (WRHI), School of Materials and Chemical Technology, Tokyo Institute of Technology, 2-12-1, Ookayama, Meguro-ku, Tokyo 152-8550, Japan; 6 Center for Physical Sciences and Technology, Saulėtekio ave. 3, LT-10257 Vilnius, Lithuania

**Keywords:** black-Si, antireflection, photoanode, water splitting

## Abstract

The fabrication and characterization of photoanodes based on black-Si (b-Si) are presented using a photoelectrochemical cell in NaOH solution. B-Si was fabricated by maskless dry plasma etching and was conformally coated by tens-of-nm of TiO_2_ using atomic layer deposition (ALD) with a top layer of CoO${}_{x}$ cocatalyst deposited by pulsed laser deposition (PLD). Low reflectivity R<5% of b-Si over the entire visible and near-IR (λ<2 μm) spectral range was favorable for the better absorption of light, while an increased surface area facilitated larger current densities. The photoelectrochemical performance of the heterostructured b-Si photoanode is discussed in terms of the n-n junction between b-Si and TiO_2_.

## 1. Introduction

**Figure 1:**
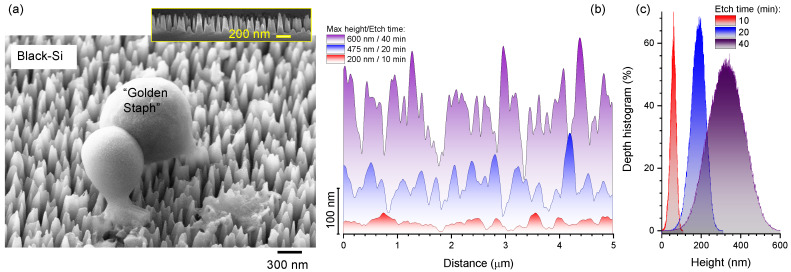
Mechanically bactericidal surface of b-Si. (**a**) Scanning electron microscope (SEM) image of a Gram-positive *Staphylococcus aureus* “Golden Staph” cell ruptured on b-Si. Inset SEM image shows the side profile of b-Si prepared by typical 20 min dry plasma etching with SF_6_ and O_2_ gases. (**b**) Height profiles of atomic force microscopy (AFM) cross sections for different plasma dry etching times of 10, 20, and 40 min (see Appendix for SEM micrographs). (**c**) Depth histogram: a depth (height) pixel distribution in the AFM image of b-Si etched for different times (see Appendix A for the bearing ratios and statistical analysis).

The nanotexturing of silicon (Si) to endow the surface topography with a random array of high-aspect-ratio spikes that can efficiently trap light (enhancing absorption, since reflection is suppressed) due to a gradual refractive index change—known as b-Si [1]—has demonstrated a wide range of useful properties: b-Si acts as an anti-reflection surface over the VIS-IR spectral range, exhibits efficient bactericidal activity towards gram-positive and gram-negative bacteria [2] (Figure 1a), can rupture the soft membranes of red blood cells [3], acts as a field emitter electrode [4], can be used as a substrate for surface enhanced Raman sensing/spectroscopy (SERS) [5], and has application in solar cell technology [6,7]. Additionally, the optical binding/trapping of small particles on b-Si was recently demonstrated at very high laser powers when the nonlinear optical effects inside the trapped material could be induced [8]. Furthermore, the high thermal conductivity of b-Si, due to its crystalline nature, is a useful property in heat dissipation aided by the large surface area of b-Si. This wide range of applications are enabled by the specific physico-chemical properties of the nanostructured Si surface.

There is increasing interest to use b-Si as a photoelectrode in the photelectrochemical (PEC) splitting of water as both a photoanode and photocathode. Nanoporous p-type b-Si with a hybrid organic/inorganic interfacial architecture, consisting of an organic molecular monolayer beneath ALD deposited TiO_2_ modified with Pt nanoparticles (NPs), was recently studied as a photocathode [9]. The critical importance of the careful consideration of the semiconductor junction engineering was pinpointed. Yang et al. (2019) introduced a dual protection layer strategy where the first layer of TiO_2_ was deposited on p-type nanoporous b-Si, while the second layer was deposited on top of the hydrogen evolution reaction (HER) co-catalyst [10]. Significant improvement in the durability of b-Si photocathodes in both acid and alkaline medium was demonstrated. Contrary to these results, Oh et al. (2019) reported that transition metal NPs electrodeposited directly onto n-type b-Si surfaces can stabilize photoanodes without the need for a protection layer [11]. However, the role of the protective layer in their study was, in fact, played by thermally grown SiO${}_{x}$. Recently, b-Si demonstrated enhanced photoelectrochemical efficiency and stability using a conformal TiO_2_ film on a nanoporous n-type silicon photoanode [12]. This b−Si/TiO_2_/Co(OH)_2_ nanostructured photoelectrode produced a saturated photocurrent density of 32.3 mA cm${}^{-2}$ at an external potential of 1.48 V versus reference electrode (RHE) in 1 M NaOH electrolyte under 1 Sun illumination. The authors demonstrated that an amorphous ALD TiO_2_ layer was able to passivate defective surface sites and increase the efficiency of electron-hole separation as well as the lifetime of minority charge carriers in n-type b-Si PEC photoelectrodes.

Here, b-Si surfaces are assessed for their photoelectrochemical (PEC) performance by exploring the influence of surface spike topography (height/pitch) and TiO_2_ film thickness. A layer of CoO${}_{x}$ as oxygen evolution reaction (OER) cocatalyst is explored to enhance the photo-current output.

## 2. Experimental

### 2.1. Fabrication of Photoanodes

B-Si was fabricated using n-type As-doped 100 mm diameter silicon wafers with specific resistivity of 0.005 Ω·cm and processed with a Samco RIE101iPH inductively coupled plasma (ICP) assisted reactive ion etching (RIE) tool. Si surfaces were initially cleaned with isopropanol and then dried under nitrogen gas flow to remove contaminants. Si wafers were then etched according to the following recipe: the etchant gases were SF_6_/O_2_ with respective flow rates of 35/45 sccm. The process pressure was 1 Pa, ICP power of 150 W, and RIE bias power of 15 W. Etching time was varied between 10 and 40 min to produce high aspect ratio nanopillars of differing heights (Figure 1 and Figure A2). During the etching process, the spontaneous passivation mask was not efficiently removed during alternating rounds of etching and deposition, therefore a simple 10 wt% sulfuric acid solution and sonication for 10 min was used to remove the contaminative mask. Figure 1 shows a typical surface pattern measured by scanning electron microscopy (SEM) and height profiles obtained by atomic force microscopy (AFM).

### 2.2. TiO_2_ and CoO${}_{x}$ Cocatalyst Deposition

**Figure 2:**
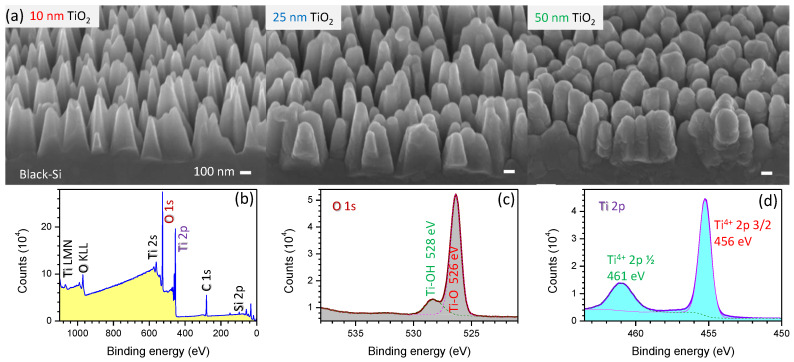
Surface characterization of b-Si photoelectrodes. (**a**) Side-view SEM images of b-Si coated with different thickness of TiO_2_ by atomic layer deposition (ALD). Scale bars 100 nm. b-Si was etched for 40 min. (**b**–**d**) XPS surface analysis for wide and narrow spectral windows at O1s and Ti2p bands from b-Si sample coated with 25 nm of TiO_2_.

TiO_2_ thin films of 10, 25, or 50 nm were deposited using atomic layer deposition (ALD) (Cambridge Nanotech ALD Fiji F200 & Savannah s100). B-Si samples were used for the ALD coating without the removal of native oxide. Figure 2 shows SEM images and the X-ray photo-electron spectroscopy (XPS) characterization of the surfaces after TiO_2_ deposition. A cobalt oxide CoO${}_{x}$ cocatalyst layer was deposited using a pulsed laser deposition (PLD) setup based on a 248 nm wavelength KrF eximer laser [13]; PLD is a practical method to deposit materials from targets with complex composition at a fast deposition rate. The resultant surface morphology was inspected by SEM.

### 2.3. PEC Measurements

PEC measurements were performed in a three-electrode configuration in 0.5 M NaOH (pH 13.0) aqueous solution. The b-Si photoelectrode was used as the working electrode. Silver paste was applied for the electrical connection between the sample and the clamped wire; an epoxy resin covered the contact and insulated it from the electrolyte. Epoxy resin was also used to insulate the back side and the edges of the b-Si sample to avoid an electrical short-circuit with only the front side of the sample exposed to the electrolyte and light. A coiled Pt wire and Ag/AgCl were used as the counter and reference electrodes, respectively. To simplify the display of the working electrode potential, the potential versus reference hydrogen electrode (RHE), obtained according to the relation E (vs. RHE) = E (vs. Ag/AgCl) + 0.197 + 0.059pH, is adopted throughout this article. A Solartron 1286 electrochemical interface was used to carry out the voltage scan and current collection. Potentiodynamic measurements with a scan rate of 10 mV/s in the potential window of 0.5–1.7 V RHE were performed to investigate the PEC performance of b-Si-TiO_2_ multiphases. The chopped dark-light current densities were normalized according to the illuminated area. The light source was a 150 W Xe lamp equipped with an AM 1.5 G filter (100 mW/cm${}^{2}$, Newport 66477-150XF-R1) calibrated with a photodetector (Gentec-Electro Optics, Inc., Quebec City).

## 3. Results and Discussion

B-Si has antireflective properties across the entire visible spectrum range [14,15] with a reflectivity of only a few percent R<5% (Figure A2) after 20 min of plasma etching. Since Si can be either an n-/p-type electrode it was interesting to explore application of b-Si as a photoelectrode with more efficient light absorption. Furthermore, an increase of the surface area, due to nanotexturing [16], is another advantage of considering b-Si for a large current operation of photo-electrochemical cells. Complex pathways of (photo)electrochemical modification of Si are possible in acidic solutions, due to the different valence states of Si [17]. In basic solutions, Si is usually stable at room temperature [18], hence, is promising as an electrode. When metals or semiconductors are deposited over b-Si surfaces, a light scattering pattern that creates the de-polarization of light and the E-field component perpendicular to the electrode or catalyst surface can be formed and facilitate a charge (electron or hole) transport through the interface [19].

### 3.1. Black-Si with TiO_2_: Potentiostatic and Potentiodynamic Scans

**Figure 3:**
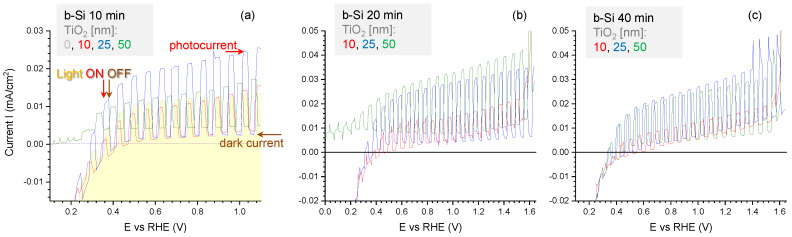
Potentiodynamic scans for the b-Si photoanodes fabricated for (**a**) 10, (**b**) 20 and (**c**) 40 min and coated with 10, 25, or 50 nm TiO_2_, respectively, in NaOH electrolyte under chopped light illumination; x, y-scales are different for a better presentation.

The potentiodynamic polarization curves recorded in the solution of 0.5 M NaOH under chopped illumination for b-Si electrodes fabricated using different etching times and coated with TiO_2_ layers of different thickness are compared in Figure 3. A slight increase in the magnitude of dark currents with increase in etching duration can be observed in Figure 3a–c. This can be attributed to the increase in the electrochemically active surface area of the electrode with etching time, which is consistent with the height profiles of AFM cross-sections shown in Figure 1b. Another observation is that the deposition of TiO_2_ on b-Si leads to an increase in photoanodic current. This is especially obvious in the case of b-Si samples with higher roughness, i.e., etched for 20 and 40 min. The most significant effect of TiO_2_ on the photocurrent is observed when the TiO_2_ thickness is increased from 10 nm to 25 nm, whereas a further thickening of the layer (up to 50 nm) was not efficient in terms of the photoelectrochemical performance of the samples.

**Figure 4:**
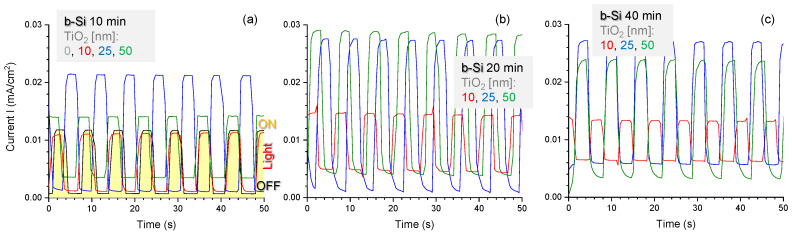
Potentiostatic scan at 1.23 V vs. RHE for the b-Si photoanodes fabricated for (**a**) 10, (**b**) 20 and (**c**) 40 min and coated with 10, 25, or 50 nm TiO_2_, respectively, in NaOH electrolyte under chopped light illumination.

**Figure 5:**
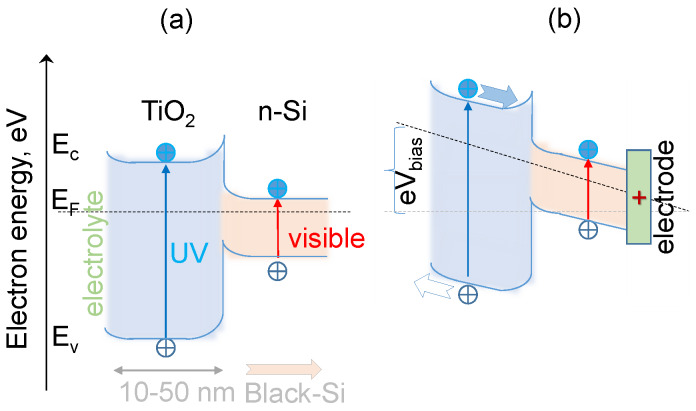
Band diagram of n-n junction between Si and TiO_2_ without (**a**) and with (**b**) bias. B-Si was fabricated using n-type Si. Under positive bias, holes drift to the titania-solution interface while electrons are drawn into the electrode bulk. The bandgap of TiO_2_ is 3.1 eV (400 nm) and 1.12 eV for Si. Under a white light (spectrally broad) illumination, e-h generation occurs at both the materials forming a TiO_2_-Si n-n-junction. Arrows show e, h drift direction.

The same regularities can be seen in chronoamperograms run under chopped illumination at the stationary potential of 1.23 V (Figure 4). The highest photocurrents (taken as the difference between the current values in the dark and under illumination) were observed in the case of b-Si samples etched for 20 min with 25 nm thick TiO_2_ layer (Figure 4b), which means that such fabrication conditions ensure the most efficient generation, separation, and transport of the charge carriers. To analyze these processes in more detail, an energy band diagram of n-n junction between n-type b-Si and n-type TiO_2_ has been constructed and is shown in Figure 5. These semiconductors form a type-I, or straddling gap, heterojunction. In accordance with their bandgap values, i.e., 3.1 eV for TiO_2_ and 1.12 eV for Si, titania will absorb the UV portion of incident light, while silicon will absorb longer wavelengths. In the case of an n-type photoanode immersed in electrolyte solution under illumination (white light), photogenerated holes should be drifting to the semiconductor-electrolyte interface to participate in the oxidation of solution species (water molecules), whereas photoelectrons should be driven to the electrode bulk and towards the counter electrode to take part in the reduction reaction. Due to the upward band bending at the heterojunction between the two n-type semiconductors, an energy barrier is formed for the transport of photogenerated electrons from the conduction band of TiO_2_ to that of Si (Figure 5a) at no external bias conditions. The barrier for photoholes drifting from the valence band of Si to the valence band ofTiO_2_ is even higher. The positive polarization of the electrode provides the energy required to overcome this barrier (Figure 5b) and this is the reason why the photocurrent increases with an increase in the electrode potential, as can be seen in Figure 3.

The surface area increase with etching time *t* can be evaluated from the height evolution of the b-Si pyramids as h(t) [15]. The side walls of the b-Si pyramids have an incline of $\sim 6^{{}^{\circ}}$ normal to the initial flat surface of Si. The surface area of b-Si can be estimated using a scaling argument by measuring the side-length $x{\left(t\right)}^{2}$, where *x* is measured from a b-Si cross-section (inset Figure 1a, Figure A2). For example, a linear evolution of the height was established following h(t)/h(15 min)=4.5×t/15 min, where h(15 min) is the height of the pyramids following a 15 min etch. The side-length of the b-Si cross-section after $t_{1}=15$ min etching was increased by *x*(15 min)/*x*(0 min) ≈3.96, hence the total surface area increase can be estimated as $x^{2}\propto 15.7$ times. For longer etching times $x{\left(t\right)}^{2}\propto \sqrt{h\left(t\right)/h\left(t_{1}\right)}x{\left(t_{1}\right)}^{2}$, where $t_{1}=15$ min. After 45 min etching, h=900 nm tall pyramids of b-Si were formed [15] and the surface area was approximately 30 times larger, as compared to the initial flat surface of Si.

### 3.2. Black-Si with TiO_2_ and CoO${}_{x}$: Potentiostatic and Potentiodynamic Scans

**Figure 6:**
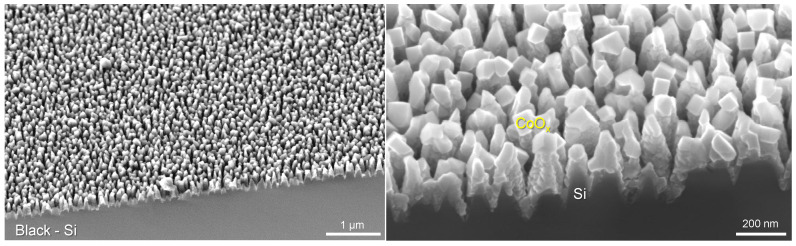
SEM images of as-fabricated b-Si photoanodes etched for 20 min and coated with 10 nm TiO_2_ with addition of 150 nm CoO${}_{x}$ cocatalyst.

Semiconductor modification with oxygen/hydrogen evolution (OER/HER) cocatalysts is a common strategy to increase the efficiency of photoelectrochemical water splitting reactions [20]. Cobalt oxide-based materials have demonstrated themselves as a promising candidate for the development of non-noble metal oxygen evolution catalysts. Non-noble metals are of interest to improve the relatively high overpotential and slow kinetics of the OER [21]. In this study, PLD was used to deposit 150 nm of cobalt oxide CoO${}_{x}$ on b-Si surfaces etched for 20 min and coated conformally with 10 nm TiO_2_ by ALD. The surface morphology of the CoO${}_{x}$ cocatalyst layer on b-Si is shown in Figure 6. The deposition of materials of 10–100 nm thickness over the nano-rough surface of b-Si by different methods (sputtering, e-beam or thermal evaporation) do not normally block the surface and the high surface area and nano-roughness which decreases reflectivity is maintained.

The presence of cobalt oxide crystallites on the surface of b-Si/TiO_2_ spikes is clearly recognizable (Figure 6). It is important to note that the fabrication conditions adopted in this study for the formation of a heterostructured photoelectrode allowed the preservation of the initial morphology of the b-Si substrate, contrary to the case reported in ref. [12], where the top layer of Co(OH)${}_{2}$ was electrodeposited on b-Si/TiO_2_ (8 nm) blocking large areas of underlying substrate beneath a thick coating.

**Figure 7:**
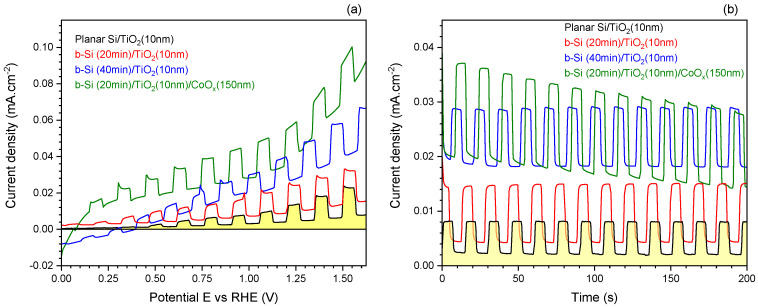
Potentiodynamic (**a**) and potentiostatic (**b**) scan at 1.23 V vs. RHE for the b-Si photoanodes fabricated for 20 and 40 min and coated with 10 nm TiO_2_ with addition of 150 nm CoO${}_{x}$ cocatalyst layer in NaOH electrolyte under chopped light illumination. The potential scan speed was 10 mV/s and light ON-OFF chopping with ~8 s period in (B).

The results of the PEC investigations are summarized in Figure 7 and compared with the response of planar Si photoelectrode with 10 nm TiO_2_ coating as well as that of b-Si fabricated for 40 min with 10 nm TiO_2_. The increase in both dark- and photo-currents in the sequence: planar Si/TiO_2_ (10 nm) < b-Si(20 min)/TiO_2_(10 nm) < b-Si(40 min)/TiO_2_(10 nm) should be attributed to an increase in the electrochemically active surface area of the electrode (Figure 7a). It should be noted, however, that an increase in dark currents is more pronounced than that of photocurrents (taken as the difference between the current values in dark and under illumination). Moreover, the increase in both dark- and photo-currents of planar-Si vs. b-Si is not comparable with the increase in surface area of the samples (about 30 times), as discussed above. A possible explanation for this could be the previously discussed energy barrier that forms at the heterojunction between n-type Si and TiO_2_ (Figure 5). The deposition of CoO${}_{x}$ leads to a significant increase in photocurrents, especially in the range of lower potential values, i.e., 0.1 V < *E* < 1.1 V. This can be attributed to efficient hole-mediated oxidation of water molecules to O${}_{2}$ on the surface of the cobalt oxide cocatalyst.

**Figure 8:**
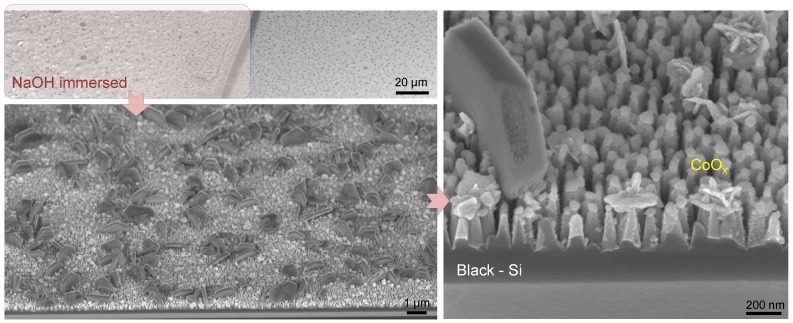
SEM images of b-Si photoanodes etched for 20 min and coated with 10 nm TiO_2_ with addition of 150 nm CoO${}_{x}$ cocatalyst after the exploitation for 30 min in photoelectrochemical cell.

Chronoamperograms recorded under chopped illumination at the stationary potential of 1.23 V (Figure 7b) reveal the same general trends described above. A slight decay of the PEC response of CoO${}_{x}$-modified b-Si(20 min)/TiO_2_(10 nm) sample can be observed during the 200 s long measurement at 1.23 V. This points to some rearrangement of the photoactive layer. SEM micrographs of the photoelectrode following the OER-exploitation in the PEC cell for 30 min in NaOH electrolyte (Figure 8) reveal drastic changes in the morphology of the sample with the formation of sub-micrometer sized hexagonal crystals randomly distributed all over the surface, characteristic of Co(III)OOH [22], which is known to play the role of active site in oxygen evolution reaction [23] and is in agreement with other studies showing the in situ formation of Co(III)OOH [24,25].

The mechanism of photoelectrocatalytic OER on the surface of CoO${}_{x}$-modified b-Si(20 min)/TiO_2_(10 nm) could be as follows: holes photogenerated in b-Si/TiO_2_ drift to the electrode/solution interface, where they are captured by CoO${}_{x}$. This interaction leads to oxidation of active cobalt oxide sites to Co(IV), the formation of which, during photocatalytic water oxidation, has been previously evidenced [26]. The authors suggested that two sequential hole injections convert the adjacent Co(III)–OH groups to Co(IV)=O centers. Subsequent addition of the water molecule to one of the oxo sites leads to O-O bond formation, followed by reduction of both cobalt centers to Co(III) and catalyst recovery. Such catalytic oxidation/reduction cycle explains the recrystallization/restructuring of CoO${}_{x}$ layer seen in SEM images of the sample which underwent photoelectrochemical testing (Figure 8). Irrespective of the rearrangement of cocatalyst layer during PEC performance, the spiky morphology of b-Si is retained, which means that ALD-deposited conformal TiO_2_ coating effectively protects silicon substrate from chemical dissolution in alkaline medium.

When considering the semiconductor/electrocatalyst interface, it is interesting to note that significantly higher photocurrents were reported when Co(OH)${}_{2}$ was electrodeposited on b-Si with 8 nm ALD of TiO_2_ [12], or when a continuous conformal 50 nm CoO${}_{x}$ layer was deposited via ALD on planar n-type Si with 2 nm SiO${}_{x}$ [25]. In our work, the coating of b-Si surface with CoO${}_{x}$ via PLD was not sufficiently bulky to cover the substrate with islands of cocatalyst as compared to Yu et al. (2017), where the authors suggested that a large coverage of cocatalyst on the photoanode is kinetically favorable for OER [12]. In contrast, Oh et al. (2019) demonstrated that photoanodes of b-Si with electrodeposited nanoparticles of Ni yielded photocurrents as high as 23 mA cm${}^{-2}$ [11]. In the latter case, special care was taken to ensure good electrical contact between Ni and Si by electroplating Ni directly onto freshly hydrogenated b-Si substrate. It is possible that the removal of native SiO_2_ could improve the photoelectrochemical performance of the nano-heterostructure fabricated herein. Thus, the comparison of results reported on b-Si photoelectrodes is not straightforward, since many aspects have to be considered and taken into account [27], and the understanding of the semiconductor/catalyst interface needs to be advanced in order to properly engineer and optimize the performance of water-splitting devices.

## 4. Conclusions and Outlook

B-Si passivated with TiO_2_ nano-layers deposited via ALD can be used as photoanodes. Such surfaces can be utilized for the deposition of the co-catalyst of water oxidation to drive OER. Structural characterization of the b-Si photoanodes reveal their nano-roughness which is instrumental for increase of surface area (current density) and reduction of reflectivity *R* (increase of absorbance A=1−R−T where *T* is the transmittance). The experiments were carried out at 1.23 V potential, which is the thermodynamic potential of water oxidation to O${}_{2}$, and could produce larger photo-currents at a larger bias. This is particularly relevant for photoelectrolysis of water on TiO_2_ [28] (Figure 5) where a strong increase in the OER rate is observed at 1.77 V [29]. One further improvement of photoanodes based on b-Si could be achieved by the removal of the native SiO_2_ on Si, which is ~2 nm, by Ar-plasma treatment. Native oxide is known to cause low photo-currents in the photoelectrolysis of water and solar cells [29].

**Figure A1:**
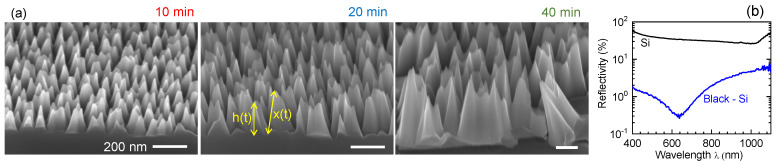
Topography and reflectivity of as-fabricated b-Si. (**a**) SEM micrographs at 45° tilt of b-Si etched for 10, 20 and 40 min, respectively. The height h(t) and side-length x(t) of nano-pyramids evolution during etch time *t* was used to evaluate surface area, see text for details. (**b**) Reflectivity of b-Si etched for 15 min was R<1% over most of visible spectral range. Reflectivity of Si (100) wafer is shown for reference. Until the bandgap energy 1.12 eV (λ=1107 nm), reflectivity $R={\left(n-1\right)}^{2}/{\left(n+1\right)}^{2}$ is dominated by the high real part of the refractive index, *n*; for normal incidence in air $n_{a}=1$.

**Figure A2:**
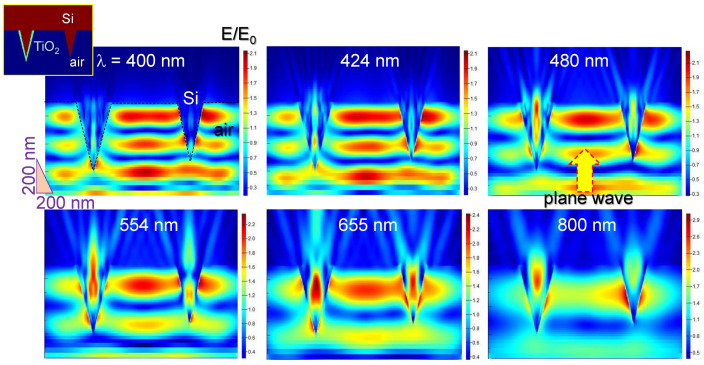
Finite differences time domain (FDTD) simulations (Lumerical) of light intensity distribution on b-Si pyramids with 10 nm TiO_2_ coating and without at different wavelengths λ. The inset shows the refractive index cross section where TiO_2_ is modelled by n=2.5 for all the wavelengths while refractive index of Si was taken from the Lumerical data base with real and imaginary parts $\tilde{n}\left(\lambda \right)=n\left(\lambda \right)+i\kappa \left(\lambda \right)$. The E-field is polarised in the plane of incidence and $E_{0}=1$.

B-Si coated with TiO_2_ is also a promising biocidal (anti-viral and bactericidal) surface, exhibiting strong oxidizing (electron removal) properties under UV light illumination that can be used to kill attaching microbes. A uniform TiO_2_ coating of 1–10 nm was not found to change the surface morphology of b-Si (Figure 2) and its bactericidal property is expected to be maintained [2] and augmented with the photo-oxidation capability demonstrated in this work. Furthermore, the photo-oxidation capabilities can be also inferred from numerical simulations of light intensity distribution (Figure A2). A surface coating of a nano-thin layer of TiO_2_ or CoO${}_{x}$ will cause a stronger absorbance inside the b-Si needles due to to reduced reflectivity (Figure A1 and Figure A2). Thin surface layers can also contribute to a better charge separation and hole transport to the surface where the oxidation of water (by bacteria or virus) is taking place. Wurtzite CoO${}_{x}$ is a p-type semiconductor with strong absorption of photons at 0.7–1.0 eV, which is considerably smaller than the direct bandgap energy of 1.6 eV [30]. A photoanode with CoO${}_{x}$ on b-Si would harvest longer wavelengths at the surface while the positive bias of b-Si contribute to e-h separation by electron extraction (see Figure 5). A self-similar 3D fractal Si fabrication with an Hausdorff dimension of D=2.322 was demonstrated using anisotropic etching of Si [31] (see Appendix A for the statistical and fractal definitions of AFM scans). Complex fractal 3D geometries with high porosity and surface area with conformal deposition by ALD are very promising for applications in light harvesting and photo-catalysis.

## Figures and Tables

**Figure A3:**
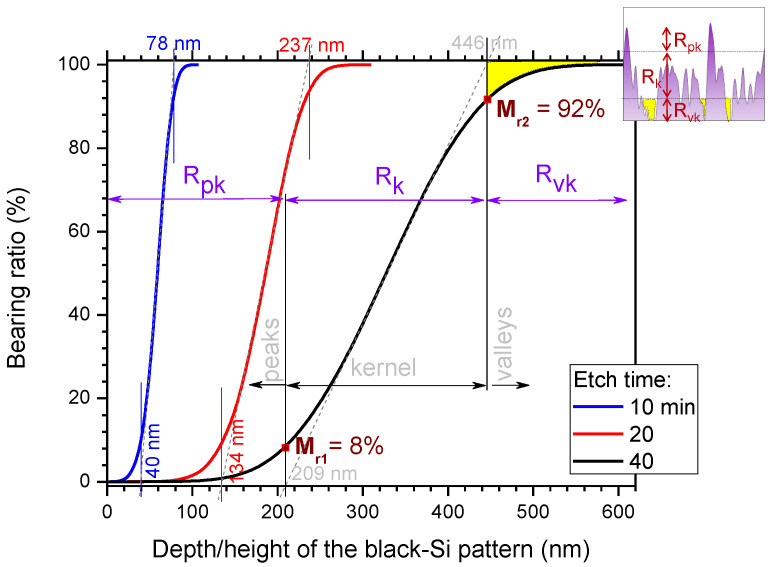
Bearing ratios of AFM traces (Figure 1b,c) of b-Si etched for 10, 20 and 40 min. See text for definitions of the parameters marked on the the Firestone–Abbott bearing curve (standards DIN4776; STN ISO 13 565-2). Inset illustrates schematically the peak, kernel, and valley extent on actual AFM profile of 40 min etched Black-Si; yellow area-mark corresponds to the air pockets in the b-Si pattern which have height (depth) extent of $R_{vk}$.

## Appendix A

Figure A2 shows the exact numerical solution of Maxwell’s equations for the plane wave incidence on b-Si pyramids. For comparison, one of the pyramids (left) is conformally covered by 10-nm-thick walls of TiO_2_. Even such a thin TiO_2_ layer acts as an anti-reflection coating and creates higher light intensity $E^{2}$ inside the Si nano-pyramids. The anti-reflection property of TiO_2_ would be even more pronounced in water solution n≈1.3 at visible wavelengths as compared with air n=1.

Figure A3 shows the Firestone–Abbott (F-A) bearing curves of b-Si etched for different etch times. The F-A curve is the cumulative probability density function of the surface profile’s height which is calculated by integrating the profile of the AFM trace. Each curve can be separated into the peak, kernel and valley sections after linear fit (tangential section) of the central part of the bearing ratio (see the marking for the 40 min etched sample). The positions of the minimum and maximum depth/height of the linear section of the AFM scan at intersections at bearing ratios of 0 and 100%, respectively, defines the kernel Section (209 and 446 nm in Figure A3). At those depth (height) values, the actual bearing ratios reads $M_{r1}=8\%$ and $M_{r2}=92\%$, which are the mass ratios (DIN4776 standard). The area below the F-A curve, up to the point $M_{r1}$, represent the mass (an integral) of the most protruding peaks of surface roughness (the peaks region). The mass ratio point $M_{r2}$ defines the point, which excludes the mass of the most deep valleys (the valleys region). The kernel roughness depth $R_{k}$ is the thickness of the kernel at the flattest part of the bearing curve with the largest increase of material under the bearing curve. The reduced peak height $R_{pk}$ and the reduced valley depth $R_{vk}$ corresponds to the thickness of the bearing curve above (below) the central kernel profile, respectively. These parameters can be defined for surfaces etched for different times (Figure A3) and lay the foundation for statistical analysis of different surfaces.

The depth (height) profile is self-similar when roughness is investigated at different scales, hence is self-affine [32]. The Weierstrass-Mandelbrot (W-M) function is used for characterization of the height profile z(x) along scan in x-direction. The W-M function has a fractal dimension, *D* [32]:

(A1) $$z\left(x\right)=G^{\left(D-1\right)}\sum_{n=n_{l}}^{\infty }\frac{\cos(2\pi \gamma^{n}x)}{\gamma^{n(2-D)}};\;1<D<2;\;\gamma >1,$$

where *G* is a scaling constant, $\gamma^{n}\equiv 1/\lambda_{n}$ is the frequency mode corresponding to the reciprocal of roughness wavelength $\lambda_{n}$; the low cut-off frequency is defined by the length *L* (AFM scan length here) as $\gamma^{n_{l}}=1/L$. The fractal dimension $D=\frac{\log N}{\log m}$, where *m* is the magnification and *N* is the number of self-similar segments 1/m, which form a line of unity length under magnification *m* ((1/m)×m=1); a one-dimensional linear segment is considered here as an example).

The fractal dimension *D* can be found from the power spectrum of z(x), which is superimposed of infinite number of frequency modes and defines the amplitude of the roughness profile at different scales (AFM profile is considered here) [32]:

(A2) $$S\left(\omega \right)=\frac{G^{2(D-1)}}{2\ln\gamma }\cdot \frac{1}{\omega^{\left(5-2D\right)}}.$$

If the power spectrum S(ω) is known, it is possible to calculate statistical parameters of the surface: the variances of the height, the slope and the curvature at different frequencies.

Another practical method to define the fractal dimension is based on the auto-correlation of AFM image (trace) [33]: R(m,n) where m,n are integers describing a shift in x and y-directions, respectively, of the original image z(x,y) which is made in *N* data acquisition steps. Then the structure function is defined as $S\left(m,n\right)=2S_{q}^{2}\left(1-R\left(m,n\right)\right)$, where $S_{q}$ is the root mean square roughness. Then the structure function of a self-similar pattern along the chosen direction τ follows $S\left(\tau \right)=K\tau^{2(2-D)}$ law [33], where *K* is the pseudo-topothesy (defines the absolute amplitude of spatial frequencies) and *D* is the fractal dimension. Nanoscale self-similar surface roughness from 20 nm to 300 nm (min-max height) was demonstrated on a thermally rescalled polystyrene coated with gold [34]. By changing scale of SEM images, self-similarity and fractal nature of such surfaces was evidenced.

The box-counting method applied to AFM or SEM images is one of the simplest ways to estimate fractal dimension [35]. The number of boxes $N\left(r\right)\propto r^{-D}$ of size *r* required to cover a chosen feature in the image provides direct estimate of the fractal dimension *D* from the slope when log-log plot is made N(r) vs. *r*. A systematic structure analysis by bearing ratio and fractal dimension of nanostructures is urgently required for understanding their link with the biocidal (anti-viral and antibacterial) properties of such surfaces, e.g., b-Si [2].
